# Do “Black individuals” really display no linguistic markers of depression?

**DOI:** 10.1073/pnas.2410997121

**Published:** 2024-07-08

**Authors:** Julian De Freitas

**Affiliations:** ^a^Marketing Unit, Harvard Business School, Boston, MA 02163

Rai et al. (1) report novel evidence that depression cannot be predicted from the Facebook posts of a sample of Black individuals but can for White individuals. Their main proffered explanation is that “depression may not manifest in language for Black individuals”. It is important, however, to acknowledge that their results may say more about their context of study than the general lack of expressions of depression in the language of Black individuals.

Their setting—posts on social media—is highly public. Many users of social media tend to not disclose their true feelings because they are wary of social judgment (2), most stay silent on controversial issues rather than actively participate (1), and many adults still believe it is taboo to disclose mental illness (3). Because depression in Black Individuals can also intersect with fraught social dynamics like prejudice, discrimination, and racism (4), we would expect them to reserve expressions of mental illness for more private settings.

Social media posts also include little context, providing less predictive power for machine learning models to detect mental illness from them. In contrast, the types of settings where people typically talk about mental health—everyday conversations, text messages, in-person and app-based therapy—provide richer, longer contexts that can improve this predictive power. Further, today’s apps for mental health and loneliness increasingly utilize AI chatbots, which might further encourage mental health disclosure given that chatbots are seen as less socially judgmental (5).

Greater context is important not only for the data inputs for classification but also for how the models are trained. While the BERT embedding model employed by the authors is exclusively trained on BooksCorpus (800M words) and English Wikipedia (2.5B words) (6), today’s state-of-the-art generative AI models are trained on hundreds of billions of words sourced from an extensive range of naturalistic datasets across the internet (7). These newer models have such sophisticated contextual knowledge that they require little training data to be fine-tuned for new classification tasks (8). Such models might similarly have a better contextual understanding of Black-produced texts, perhaps in part because they have seen more such texts in training.

At the same time, we have found that many algorithm-powered apps respond more appropriately to mental health problems that are expressed explicitly (e.g., “I want to cut myself”) than vaguely (e.g., “I just want to suffer” or “Do you ever think about cutting yourself?”) (9). This suggests the need to exercise caution in drawing conclusions about the nonexistence of Black linguistic expressions of depression based on today’s machine learning models alone.

Finally, while the authors raise the need for “expanding this research to other racial subgroups”, it is also important to acknowledge that there is considerable variation among Black Americans themselves across dimensions like educational attainment, socioeconomic status, family structure, cultural identity, and reactions to racism (10). In order to deepen understanding across this variation, future studies of Black expressions of mental illness can prudently sample, characterize, and contextualize their participants.
